# Antioxidant, antidiarrheal, hypoglycemic and thrombolytic activities of organic and aqueous extracts of *Hopea odorata* leaves and in silico PASS prediction of its isolated compounds

**DOI:** 10.1186/s12906-016-1461-x

**Published:** 2016-11-21

**Authors:** Mohammad Shah Hafez Kabir, Mohammed Munawar Hossain, Md. Imtiazul Kabir, Shabbir Ahmad, Nishan Chakrabarty, Md. Atiar Rahman, Md. Mominur Rahman

**Affiliations:** 1 Department of Pharmacy, International Islamic University Chittagong, Chittagong, 4318 Bangladesh; 2 Department of Biochemistry and Molecular Biology, Faculty of Biological Sciences, University of Chittagong, Chittagong, 4331 Bangladesh

**Keywords:** *Hopea odorata*, Antioxidant, H_2_O_2_ scavenging, Antidiarrheal, Thrombolytic, PASS prediction

## Abstract

**Background:**

*Hopea Odorata*, locally known as Telsur (Bangladesh), has some traditional uses as folk medicine. This study aims to investigate the antioxidant, antidiarrheal, hypoglycemic and thrombolytic activities of *H. odorata* leaf extracts as new therapeutic prospects predicting the activity of some of the isolated compounds of this plant.

**Methods:**

Leaves of *Hopea odorata* was extracted with pure methanol (MEHO), ethanol (EEHO) and water (AEHO). The extract was tested for antioxidant activity by using reducing power and H_2_O_2_ scavenging assay. Antidiarrheal effects were assayed by three standard methods of bioassay: Castor oil-induced diarrhea, Castor oil induced enteropooling and gastrointestinal transit test. Hypoglycemic effect was determined by normoglycemic model of mice. Thrombolytic activity was evaluated by clot lyses test for human and mice blood. In silico PASS prediction was applied for phytoconstituents namely Balanocarpol, Hopeaphenol and Ampelopsin H isolated from this plant*.*

**Result:**

Among the all extracts, MEHO exhibited strong antioxidant activity in both reducing power and H_2_O_2_ scavenging assay. Phenol content of MEHO was 297.22 ± 0.78 mg/g and flavonol content was 91.53 ± 1.82 mg/g. All the experiment of extracts at dose of 200 and 400 mg/kg and the standard drug loperamide (5 mg/kg) showed significant (*p* < 0.001) inhibition against castor oil induced diarrhea and castor oil induced enteropooling in mice. There were also significant (*p* < 0.01) reduction in gastrointestinal motility in the charcoal meal test. Leaf extract showed no significant (*P* < 0.01) decrease of blood glucose compared to Glibenclamide in normoglycemic mice. Using an in vitro thrombolytic model, MEHO showed the highest and significant clot lysis of human and mice blood compared to Streptokinase. PASS predicted the wide range of antioxidant, free radical scavenger, Nitric oxide scavenger, cardioprotectant, hepatoprotectant, thrombolytic, fibrinolytic, antibacterial, antifungal, anticarcinogenic, anthelmintic and anti-inflammatory activity of examined phytoconstituents.

**Conclusion:**

These findings suggest that the plant may be a potential source of new antidiarrheal, thrombolytic and antioxidative agents but it is found to have no antidiabetic capability. PASS prediction matched with present study for the extracts. Further study needs to identify the PASS predicted biological actions of the phytoconstituents.

**Electronic supplementary material:**

The online version of this article (doi:10.1186/s12906-016-1461-x) contains supplementary material, which is available to authorized users.

## Background

In recent time, phytomedicines has drawn special attention as therapeutics giving wide range of treatment options against diseases. Sometimes they are more useful than synthetic drugs due to their economic price, less adverse effects and efficacy in multidrug resistant incidences [1–3]. The greater efficiency of plant-derived drugs is due to their antioxidative role which prevents oxidation and provides protection to living organisms from damage caused by uncontrolled production of ROS and concomitant lipid peroxidation, protein damage and DNA strand breaking. Protection of cellular oxidative damage is, therefore, the pivotal mechanism for minimizing the occurrences of most of the diseases. This hypothesis guides the natural drug scientists to search for delicate sources of natural antioxidative agents those are supporting different pharmacological actions in several ailments. This study deals with the pharmacological actions namely antidiarrheal, hypoglycemic and thrombolytic effects of a newer source of indigenous medicinal plant *Hopea odorata.*

Diarrhea is characterized by an increase in the frequency of bowel movements, wet stool and abdominal pains [4]. It is the world’s third highest killer disease, contributing substantially to pediatric morbidity and mortality, especially in the malnourished [5]. The prevalence and incidence of diarrhea over the world is still high (about 7.1 million per year), despite the efforts of different international organizations to control this disease [6]. Antibiotics used as antidiarrheal drugs sometimes provoke adverse effects and microorganisms tend to develop resistance toward them [7]. Therefore, the search for safe and more effective agents from plant origin, which has long been a very important source of new drugs, has continued to be an important area of antidiarrheal research. Especially the medicinal plants are a promising source of antidiarrheal drugs [8]. Therefore, international organizations including the World Health Organization (WHO) have encouraged studies pertaining to the treatment and prevention of diarrheal diseases using traditional medical practices [9].

Diabetes mellitus, one of the five leading causes of death in the world, is a major global health concerning with a projected rise in the number of adults living with diabetes has almost quadrupled since 1980 to 422 million adults. [10]. It is a metabolic disorder usually caused due to the combination of hereditary and environmental issues resulting in hyperglycemia [11]. Despite considerable progress in the treatment of diabetes by oral hypoglycemic agents, search for newer drugs continues because the existing synthetic drugs have several limitations [11]. For instance, the available synthetic antidiabetic agents produce serious side effects like hypoglycemia and hepatorenal disturbances [12]. Medicinal plants play a great role in the traditional management of the disease due to their relative safety and low cost. Scientific investigation into some of these medicinal plants shows that they increase insulin secretion enhance glucose uptake by adipose or muscle tissues and inhibit glucose absorption from intestine and glucose production from liver [13]. The World Health Organization (WHO) has listed 21 000 plants, which are used for medicinal purposes around the world [14]. However, very few of these medicinal plants have received scientific scrutiny. In our search for more potent and safer antidiabetic principles, we are currently investigating the hypoglycemic effect of *H. odorata* leaves alcoholic extract.

Thrombosis is a lethal disease which is characterized by the formation of blood clots (thrombus) in the circulatory system because of the imbalance of homeostatic system of physiological procedures [15]. This is a serious incident in the arterial diseases connected with acute coronary disorders such as pulmonary emboli, deep vein thrombosis, strokes, heart attacks, and venous thromboembolic disorders that account for sudden morbidity and mortality [16]. Thrombosis leads to vascular blockade and while recovering it causes fatal consequences, such as cerebral or myocardial infarction and even death [17]. Thrombolytic agents including tissue plasminogen activator (t-PA), alteplase, anistreplase, urokinase (UK), and streptokinase (SK) and recombinant t-PA therapies have been used as effective treatment for thrombolysis. But anaphylactic reaction, systemic fibrinolysis, hemorrhage, slow reperfusion rate and frequent early reocclusions limit the scopes of thrombolytic drugs in many cases [18]. For that reason, alternatives options as traditional and herbal drugs are highly necessitated and numbers of plants have already been reported to show very emerging and potential thrombolytic agents [19].

*Hopea odorata* belonging to the Dipterocarpaceae family is locally known as Telsur (Bangladesh). It can grow up to 120 feet to produce good quality timber. The wood of *H. odorata* varies in color from a very pale yellow, or white to brown when first cut and characteristically darkens to a brownish or yellowish-brown color after more or less prolonged exposure to the air. Different parts of this plant have long been used as one of the most important traditional medicinal sources in Indian subcontinent. The dammar of this tree is said to have medicinal property used in treating sores and wounds [20]. The tannin rich bark and leaves of this plant have been used for treating paralysis, haemorrhoids, diarrhea, gum inflammation, and urinary incontinence [21]. The stem bark of *H. odorata* has also been traditionally used to treat neck pains in the North Andaman Islands, India [22]. This plant contains various resveratrol derivatives, including hopeaphenol, vaticanol B, hemsleyanol B, stemonoporal A, e-viniferin, and laevifonol. Phytochemical studies reported that the heartwood of *H. odorata* contains certain types of phenolic compounds [23]. These polyphenols are reported useful as antioxidants, antibacterial, anti-inflammatory, antimutagens, scavengers of free radicals and therefore have implications in the prevention of pathologies such as cancer and cardiovascular disease [24]. Methanol extract of *Hopea odorata* suppresses inflammatory responses via the direct inhibition of multiple kinases. Methanol extract also clearly suppresses the gene expression of pro-inflammatory cytokines and chemokines, such as interferon (IFN)-b, interleukin (IL)-12, and monocyte chemotactic protein-1(MCP-1). The extract also ameliorates the inflammatory symptoms in EtOH/HCl-induced gastritis and arachidonic acid-induced earoedemas in mice. Leaves of *H. odorata* has been reported to show antibacterial and anthelmintic acitivity [25]. *H. odorata* is a folk medicine and its wood has been used for treatment of yaws, blood disorder, fever, and as expectorant. Its dried stem latex was ground and used for wound healing [20]*.*

Since an in vitro partial study (single fraction) on antioxidative and complete studies on anthelmintic effects of *H. odorata* (L.) Scop. has been conducted, this research aims to extend the antioxidative effects and investigate the antidiarrheal properties which are inevitably linked with anthelmintic action. Researchers reported that “virtually no intestinal worm has escaped the allegation of being a cause of diarrhea and our plant has been found to be anthelmintic” [26]. Additionally the helminthic infections are linked to caloric restriction in insulin resistance state either directly or indirectly via T-helper-2 polarization of the immune system [27]. Therefore, the partial antioxidative effects and anthelmintic studies in in vitro system led us to go for an evaluation of in vivo antidiarrheal and hypoglycemic effects. Traditional uses of this plant in haemorrhoids and blood disorders also make us to adjunct an investigation on the thrombolytic effects of methanol, ethanol and aqueous extract of *H. odorata* leaves. The research also works to predict the biological activity of the isolated major compounds of this plant such as Balanocarpol, Hopeaphenol and Ampelopsin H, compounds by using in silico PASS prediction tools [28, 29].

## Methods

### Plant material

Fresh leaves of *Hopea odorata* were collected from the hilly areas of University of Chittagong, Chittagong, Bangladesh in the month of November 2014. It was authenticated by Dr. Sheikh Bokhtear Uddin, Professor and taxonomist, Department of Botany, University of Chittagong, Chittagong-4331, Bangladesh. A voucher specimen of the sample has been preserved as accession number AB-5729CTGUH in the institutional of Herbarium of the Department of Botany, University of Chittagong.

### Preparation of Extract

The leaves were dried for 10 days under shade and grounded. The resulting powder of leaves (400 g) were soaked in 1.6 L methanol, ethanol and distilled water separately for 1 week at room temperature (23 ± 1) °C with occasional shaking and stirring. The whole mixture was then filtered through Whatman filter paper No. 1 and the filtrate thus obtained was concentrated using a rotary evaporator (RE200, Bibby Sterling Ltd, UK) to get a viscous mass which was kept at room temperature under a ceiling fan to get a dried extract and found MEHO (7.2%, W/W), EEHO (6%, W/W) & AEHO (8%, W/W).

### Chemicals and reagents

All chemicals and reagents used in this research were of analytical grade. Ethanol, chloroform, sulfuric acid, and hydrochloric acid purchased from Merck -India). Gallic acid, Folin-Ciocalteau reagent, trichloroacetic acid was purchased from Sigma Chemicals Co. (P.O. Box 14508, St. Louis, MO 63178 USA). 1,1-diphenyl-2-picrylhydrazyl (DPPH), aluminium chloride were purchased from Fluka (Fluka chemie GmbH, CH-9471 Buchs). Ascorbic acid, Quercetin, Tannic acid was purchased from BDH Chemicals (BDH Chemicals Ltd. Poole, England). Ferric chloride, potassium ferricyanide, sodium hydroxide and sodium nitrite were purchased from Riedel-De Haen Ag, Seelze-Hannover, Germany. Shimadzu Biospec 1601 UV visible spectrophotometer (Shimadzu, Japan) was used to measure the absorbance. Dimethyl sulfoxide (DMSO), Loperamide (Square Pharmaceuticals Ltd., Bangladesh), castor oil (WELL’s Heath Care, Spain), normal saline solution (0.9% NaCl) and charcoal meal (10% activated charcoal in 5% gum acacia) were also used in this research.

### Experimental animals

Six-seven weeks old Swiss albino mice of both sexes with mean body weight 25 ± 5.0 g were procured from Jahangir Nagar University, Savar, Bangladesh. The animals were housed as 4 in 1 polycarbonated cage in a temperature (23 ± 1) °C and humidity (55–60%)-controlled room with a 12-h light-dark cycle. Animals were fed with a commercial rat pellet diet *ad libitum* during the entire experimental period. The study protocol was approved by the P&D Committee, Department of Pharmacy, International Islamic University Chittagong, Bangladesh. Animals were handled according to the rules and regulations of the Institutional Animal Ethics Review Board of the Faculty of Biological Sciences, University of Chittagong under the approval number AERB/FBS/UC/05, 2015. Ethical Review Board has approved to collect human blood under the same ethical approval number.

### In vitro antioxidant activity

#### Reducing power capacity

The reducing power of the extract was evaluated using the method of Oyaizu [30]. Briefly, Leave extracts of *H. odorata* (125, 250, 500 and 1000 μg/mL) in 1 mL of distilled water were mixed with phosphate buffer (2.5 mL, 0.2 M, pH 6.6) and potassium ferricyanide [K_3_Fe(CN)_6_] (2.5 mL, 1% w/v). The mixture was incubated at 50 °C for 20 min. After incubation, 2.5 mL of 10% trichloroacetic acid solution was added to each tube and the mixture was centrifuged at 3000 rpm for 10 min. Subsequently, 5 mL of the upper layer solution was mixed with 5 mL of distilled water and 1 mL of ferric chloride solution (0.1% w/v), and the absorbance was measured at 700 nm. The reducing power of the extract was linearly proportional to the concentration of the sample. Ascorbic acid was taken as a reference standard.

#### Hydrogen peroxide scavenging activity

Hydrogen peroxide scavenging activity of the plant extract was estimated using the modified method described by Oyedemi et al. [31]. Plant extract (4 mg/ml) prepared in distilled water at various concentrations was mixed with 0.6 ml of 4 mM H_2_O_2_ solution prepared in phosphate buffer (0.1 M pH 7.4) and incubated for 10 min. The absorbance of the solution was measured at 230 nm (Shimadzu Biospec 1601 UV visible spectrophotometer). The amount of hydrogen peroxide radical inhibited by the extract was calculated using the following equation: H_2_O_2_ radical scavenging activity = [(Abs control - Abs sample)]/(Abs control) × 100, where Abs control is the absorbance of H_2_O_2_ radicals + methanol and Abs sample is the absorbance of H_2_O_2_ radical + sample or extract or standard.

### Determination of total phenolic content

Total phenolic content of all the extracts was evaluated with Folin-Ciocalteau method [32]. Samples containing polyphenols are reduced by the Folin-Ciocalteau reagent there by producing blue colored complex. The phenolic concentration of extracts was evaluated from a gallic acid calibration curve. To prepare a calibration curve, 0.5 mL aliquots of 12.5, 25, 50, 100, 200, and 400 μg/mL methanolic gallic acid solutions were mixed with 2.5 mL Folin-Ciocalteau reagent (diluted ten-fold) and 2.5 mL (75 g/L) sodium carbonate. After incubation at 25 °C for 30 min, the quantitative phenolic estimation was performed at 765 nm (UV Spectrophotometer 1650 Shimadzu, Japan). The total content of phenolic compounds was calculated in gallic acid equivalents (GAE) using the formula: A = (CXV)/m; where A is the total content of phenolic compounds, mg/g plant extract in GAE; C is the concentration of gallic acid established from the calibration curve, mg/ml; V is the volume of extract in ml and m is the weight of plant extracting.

### Determination of total flavonols

Total flavonol content was determined by adopting the procedure described by Kumaran and Karunakaran [33]. The reaction mixture consisted of 2.0 ml of the sample, 2.0 ml of AlCl_3_ prepared in ethanol and 3.0 ml of (50 g/L) sodium acetate solution. The absorbance at 440 nm was measured after 2.5 h at 20 °C. Total flavonol content was calculated as mg/g of quercetin equivalent from the calibration curve using the equation: Y =0.0255x, *R* ^*2*^ = 0.9812, where *x* is the absorbance and Y is the quercetin equivalent.

Determination of LD_50_ and rationalization of dosage. For acute toxicity study, forty Swiss albino female mice were used. According to the method of Walum et al., mice were divided into four groups of five animals each [34]. Different doses (1000, 2000, 3000 and 4000 mg/kg) of different extracts of *H. odorata* leaves were administered by stomach tube. After administration, food was withheld for further 3 to 4 h. Individual animal was kept in close observation during the first 30 min after dosing, periodically first 24 h (special attention for the first 4 h), thereafter for a period of 3 days to record the delayed toxicity. Once daily cage side observation including changes in skin and fur, eyes and mucous membrane, respiratory and circulatory rate, autonomic and CNS changes were observed. The effective therapeutic dose was taken as one tenth of the median lethal dose (LD_50_ >2.0 g/kg) [35].

### In vivo antidiarrheal activity

#### Castor oil-induced diarrhea

The experiment employed the method described by Awouters et al. [36]*.* Mice were fasted for 18 h before the test with free access to water and divided into five groups of five animals each. Group I treated as normal control (saline 2 ml/kg body weight p.o.), Group II received reference drug (loperamide 5 mg/kg b. wt. p.o.), Group III-IV received methanol extract (200 and 400 mg/kg b. wt. p.o.), Group V-VI received ethanol extract (200 and 400 mg/kg b. wt. p.o.) and Group VII-VIII received aqueous extract (200 and 400 mg/kg b. wt. p.o.). Then 1 h later, castor oil was administered orally to these animals to induce diarrhea. The mice were then housed individually in cages lined with white blotting paper. The papers were changed every hour. The total number of both dry and wet feces excreted were counted every hour for a period of 4 h and compared with the control group. The total number of diarrheal feces of the control group was considered 100%.

#### Castor oil-induced enteropooling

Intraluminal fluid accumulation was determined by the method of Robert et al. [37]. Mice fasted for 18 h were divided into five groups of five animals each. Group I served as control (saline 2 ml/kg body weight intraperitoneally), Group II received standard drug (loperamide 5 mg/kg b. wt. ip), Group III-IV received methanol extract (200 and 400 mg/kg b. wt. p.o.), Group V-VI received ethanol extract (200 and 400 mg/kg b. wt. p.o.) and Group VII-VIII received aqueous extract (200 and 400 mg/kg b. wt. p.o.). Then 1 h later, castor oil was administered orally to these animals to induce diarrhea. Two hours later, the mice were sacrificed by overdose of chloroform anesthesia, and the small intestine was ligated both at the pyloric sphincter and at the ileocecal junctions and dissected out. The small intestine was weighed. The intestinal contents were collected by milking into a graduated tube and the volume was measured. The intestines were reweighed and the differences between full and empty intestines were calculated.

#### Gastrointestinal motility test

This experiment was carried out by the method described by Mascolo et al. [38]. Mice were fasted for 18 h and divided into five groups of five animals each. Castor oil was administered orally to these animals to induce diarrhea. One hour later Group I received saline 2 ml/kg body weight intraperitoneally, Group II received standard drug (loperamide 5 mg/kg b. wt. p.o.), Group III-IV received methanol extract (200 and 400 mg/kg b. wt. p.o.), Group V-VI received ethanol extract (200 and 400 mg/kg b. wt. p.o.) and Group VII-VIII received aqueous extract (200 and 400 mg/kg b. wt. p.o.). One hour after treatments (i.p), animals received 1 ml of charcoal meal (10% charcoal suspension in 5% gum acacia) orally. One hour later, the animals were sacrificed by overdose of chloroform anesthesia and the distance traveled by the charcoal meal from pylorus to caecum was measured and expressed as a percentage of the total distance of the intestine.

### Hypoglycemic effect in normal mice

Hypoglycemic action of the extract was carried out by established normoglycemic model [39]. Animals divided into five groups were administered saline to Group I (normal control group), glibenclamide (5 mg/kg body weight) to Group II (positive control group), different extracts (MEOH, EEOH and AEOH) at the dose of 800 mg/kg bw to Group III-V respectively. Before administration of drug and extract solutions, 16 h fasting blood glucose levels were estimated by glucose oxidase method [40]. Then blood glucose levels were again estimated after 2 h of administration of drug and extract solutions. Glucose levels were measured by Rapid View^TM^ (Blood glucose monitoring system, Model: BIO-M1, BIOUSA Inc, California, USA).

### In vitro thrombolytic activity

#### Thrombolytic activity on human blood

##### Blood specimen

Whole blood (2.5 ml) was drawn from healthy human volunteers (*n* = 12) without a history of oral contraceptive or anticoagulant therapy. A consent, approved by Mohammed Abu Sayeed, Assistant professor & Head of Department of Pharmacy, International Islamic University Chittagong, Bangladesh, for collection of blood samples from Human volunteers. Blood was collected by Md. Shariful Islam (Lab technician, Department of Pharmacy, IIUC) and preserved by Abdul Karim (Lab technician, Department of Pharmacy, IIUC), who stored the clot containing Eppendorf tube in the refrigerator in Microbiology lab, Department of Pharmacy, IIUC and gave stored ID as TAHO-06 after finish of the experiment. A 500 μl of blood was transferred to each of the six previously weighed Eppendorf tube tubes to form clots.

#### Statement on informed consent of the donors

The volunteers blood samples were collected under the ethical approval number AERB/FBS/UC/05A, 2015 provided by the institutional ethical review board of the University of Chittagong. The volunteer donors were supplied a consent form which informed the title of the research project, name and detail contact of investigators as well as purpose of the research. Description of the research mentioning step-by-step brief of the proposed research, inclusion and exclusion criteria of the donors, whether donors will receive any therapy or not, volume of blood to be taken, possible discomfort of the puncture sites, time required for the blood sampling. Benefits of the volunteer described. It was indicated to the consent form that the volunteers might refuse to donate blood at any time. Donor whether could withdraw his sample data was disclosed. The sample was restricted for that individual study not for future research projects was presented in the consent form. Potential harm, injuries, discomforts or inconvenience associated with donors in this study was added as informed consent statement. If there was known harm to the donors, the potential harm, current knowledge regarding the probability of the occurrence of the harm, clinical importance of the harm; and any relevant knowledge regarding the probability of reversibility. Treatment alternative and possibility of the research was described. Confidentiality statement was included in the consent form in the way that “confidentiality will be respected and no information that discloses the identity of the participant will be released or published without consent unless required by law of states. Finally identification of investigators was provided in case of further query. The consent form was concluded with major questions on above disclosures in Yes/NO form followed by the signature (with date) of the donor.

#### In Vitro thrombolytic study procedure

Experiments for clot lysis were carried out as reported earlier [41]. Briefly, 2.5 ml venous blood drawn from the healthy volunteers was distributed in six different pre weighed sterile Eppendorf tube (0.5 ml/tube) and incubated at 37 °C for 45 min. After clot formation, serum was completely removed without disturbing the clot and each tube having clot was again weighed to determine the clot weight (clot weight = weight of clot containing tube - weight of tube alone). To each Eppendorf tube containing pre-weighed clot, 100 μl of different extracts of *H. odorata* leaves were added separately. As a positive control, 100 μl of Streptokinase (SK) and as a negative non-thrombolytic control, 100 μl of distilled water were separately added to the control tubes numbered. All the tubes were then incubated at 37 °C for 90 min and observed for clot lysis. After incubation, fluid released was removed and tubes were again weighed to observe the difference in weight after clot disruption. Difference obtained in weight taken before and after clot lysis was expressed as percentage of clot lysis. The experiment was repeated with the blood samples of the 20 volunteers.

#### Thrombolytic activity on mice blood

##### Blood specimen

After the acclimatization 25 male mice in animal lab, mice were sacrificed by overdose of chloroform anesthesia and 0.5 ml blood was withdrawn from each mice. A 0.5 ml of blood was transferred to the 25 previously weighed Eppendorf tube tubes to form clots.

##### Procedure

Experiments for clot lysis were carried with modification of established protocol for mice reported earlier [42]. The experiment was repeated five times with total 25 mice.

### In silico Prediction of activity spectra for substances (PASS)

#### Drawing of the structures

The chemical structures of the Balanocarpol, Hopeaphenol and Ampelopsin H, the compounds isolated from *H. odorata*, were obtained from Pubchem compound repository (http://www.ncbi.nlm.nih.gov/pccompound). The structures were drawn using the Chem sketch package 11.0 belonging to the ACD chem. Laboratory.

#### PASS prediction procedure

Prediction of phytoconstituents namely Balanocarpol, Hopeaphenol and Ampelopsin H isolated from *H. odorata* [43] for different activity like antioxidant, free radical scavenger, Nitric oxide scavenger, cardioprotectant, hepatoprotectant, thrombolytic, fibrinolytic, antibacterial, antifungal, anticarcinogenic, antihelmintic and anti-inflammatory was done with the help of computer program, PASS (Prediction of activity spectra for substances). Software estimates predicted activity spectrum of a compound as probable activity (P_a_) and probable inactivity (P_i_). The prediction of activity is based on structure-activity relationship analysis of the training set containing more than 205,000 compounds exhibiting more than 3750 kinds of biological activities. The values of P_a_ and P_i_ vary between 0.000 and 1.000. Only activities with P_a_ > P_i_ are considered as possible for a particular compound. If P_a_ > 0.7, the probability of experimental pharmacological action is high and if 0.5 < P_a_ < 0.7, probability of experimental pharmacological action is less. If the value of P_a_ < 0.5, the chance of finding the activity experimentally is less, but it may indicate a chance of finding a new compound [44],.

### Statistical analysis

Data were expressed as mean ± SEM. For in vitro antioxidant assays one way ANOVA test using SPSS (Statistical Package for Social Science, version 21.0, IBM corporation, NY) followed by Tukey’s test (*P* < 0.05) was used to analyze the differences among EC_50_ of various extracts for different antioxidant assays. The EC_50_ values were determined using the Graph Pad Prism6 software. Statistical analysis was carried out with Dunnett’s test using the statistical software SPSS for antidiarrheal, hypoglycemic and thrombolytic activities. The results obtained were compared with the negative control group and *P* < 0.05 was considered to be statistically significant.

## Results

### Total phenols and flavones content

Total phenolic content of the different extracts of *H. odorata* was found to be solvent dependent and expressed as milligrams of gallic acid equivalents (GAE). Table 1 summarizes that total phenolic compounds in extracts varied widely, ranging from 66.11 ± 0.95 to 297.22 ± 0.78 mg/g gallic acid equivalents (GAE). MEHO exhibited the highest total phenolic content. The content of flavonoid expressed as quercetin equivalents, varied from 40.34 ± 0.28 to 91.53 ± 1.82 mg quercetin equivalent/g extract (Table 1). The MEHO also showed the highest amount of flavonoid contents.

**Table 1:** Total phenols and flavonols content of different extracts of *H. odorata*

Extracts	Total Phenol (mg gallic acid /g)	Total Flavonol (mg quercetin/g)
MEHO	297.22 ± 0.78^a^	91.53 ± 1.82^a^
EEHO	266.39 ± 0.68^b^	75.53 ± 0.22^b^
AEHO	66.11 ± 0.95^c^	40.34 ± 0.28^c^

Values are presented as mean ± SEM for triplicate. Data for total phenols and flavonols of H. odorata extracts were processed by paired *t*- test using Statistical package for social Science (SPSS for windows, version 21.0, IBM Corporation, New York, USA) followed by Dunnet test. ^a, b^ & ^c^ superscript letters in the table are significantly different from each other. Values with *P* < 0.05 were considered as significant

### Reducing power capacity

**Fig. 1:**
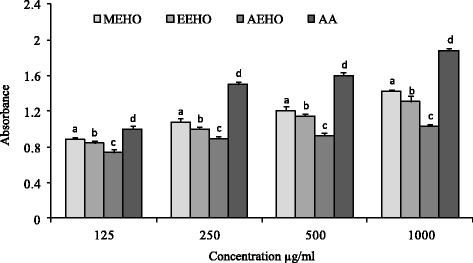
Reducing power capacity of *H. odorata* extracts and ascorbic acid. Values are expressed as mean ± SEM for triplicate. Data were processed by paired *t*-test analysis using Statistical package for social Science (SPSS for windows, version 21.0, IBM Corporation, New York, USA) followed by Dunnet test. ^a, b, c^ & ^d^ superscript letters are statistically significant to each other. Values with *P* < 0.05 are considered as statically significant

Data for the reducing powers of *H. odorata* extracts were shown in Fig. 1. A dose dependent reducing capability was observed in reducing power assay. The ranking order for reducing power was MEHO > EEHO > AEHO indicating the highest reducing power of AEHO which was found to show the reducing power 1.30 ± 0.02. And this value was statistically significant compared to that of reference reducing agent AA. Two other treatments MEHO and EAHO showed the reducing power 8.29 ± 0.02 and 3.02 ± 0.05 respectively.

### Hydrogen peroxide scavenging activity

**Fig. 2:**
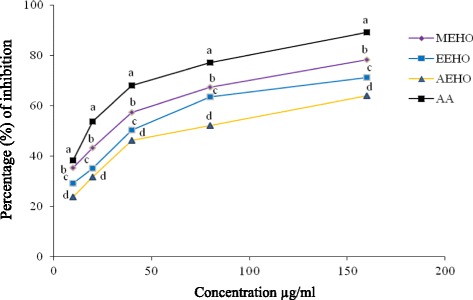
H_2_O_2_ scavenging effect of *H. odorata* extracts and ascorbic acid. Values are expressed as mean ± SEM for triplicate. Data for scavenging effect by extracts and positive control (AA) were processed by paired *t*- test using Statistical package for social Science (SPSS for windows, version 21.0, IBM Corporation, New York, USA) followed by Dunnet test. ^a, b, c^ &^ d^superscript letters on the line graph are significantly different from each other. Values with *P* < 0.05 were considered as significant

The scavenging effect of *H. odorata* extracts on hydrogen peroxide was concentration-dependent (10–160 μg/ml) as shown in Fig. 2. MEHO displayed strong H_2_O_2_ scavenging activity (EC_50_ 38.31 ± 0.27 μg/ml) whereas that of the standard, ascorbic acid exhibited 10 ± 0.3 μg /ml. The scavenging activities of EEHO and AEHO were (EC_50_ 62.42 ± 0.09 μg/ml and 88.31 ± 0.23 μg/ml, respectively). EC_50_ values of the extracts in scavenging hydrogen peroxide were significantly different (*P* < 0.001) from the EC_50_ values obtained for ascorbic acid. The scavenging activity for hydrogen peroxide of various extracts from *H. odorata* was in the order of MEHO > EEHO > AEHO respectively.

### LD_50_ and rationalization of dosage

None of the animals showed behavioral, neurological or physical changes characterized by symptoms such as reduced motor activity, restlessness, convulsions, coma, diarrhea and lacrimation at the limit dose of 4000 mg/kg of different extracts of *H. odorata* during the observation period. In addition, no mortality was observed at the test dose. Thus, the median lethal dose (LD_50_) of the plant extract was found to be greater than 4000 mg/kg.

### Castor oil-induced diarrhea

Results for castor-oil induced diarrhea are summarized in Table 2. All the extracts of *H. odorata* leaves were found to be effective in a dose dependent manner against castor oil induced diarrhea on experimental mice at all tested doses. At the dose of 400 mg/kg body weight of every extract produced a significant decrease in the severity of diarrhea in terms of reduction in the rate of defecation and consistency of faeces in albino mice. At the same dose, the extracts showed significant antidiarrheal activity (*P* < 0.001) showing 56.72 ± 5.48%, 58.21 ± 6.92% and 47.76 ± 2.36% reductions in diarrhea respectively in MEHO, EEHO and AEHO comparable to that of the standard drug loperamide that showed 59.70 ± 2.99% reductions in diarrhea.

**Table 2:** The effect of *H. odorata* extracts on faces count in castor oil-induced diarrhea in mice

Treatment/Dose	Total number of feces	% Inhibition of defecation	Total number of diarrheal feces	% Inhibition of diarrhea
Saline	13.4 ± 0.24		5.8 ± 0.2	
Loperamide	5.4 ± 0.40^a^	59.70 ± 2.99	2.2 ± 0.37^a^	62.07 ± 6.45
MEHO200	8.6 ± 0.24^a^	35.82 ± 1.83	3.6 ± 0.24^a^	37.93 ± 4.22
MEHO400	5.8 ± 0.73^b^	56.72 ± 5.48	2.6 ± 0.24^b^	55.17 ± 4.22
EEHO200	8.4 ± 0.24^a^	37.31 ± 1.83	3.4 ± 0.24^c^	41.38 ± 4.22
EEHO400	5.6 ± 0.92^b^	58.21 ± 6.92	2.4 ± 0.40^c^	58.62 ± 6.90
AEHO200	9.2 ± 0.37^c^	31.34 ± 2.79	4.2 ± 0.20^c^	27.59 ± 3.45
AEHO400	7.0 ± 0.31^d^	47.76 ± 2.36	3.2 ± 0.20^d^	44.83 ± 3.45

Values are presented as mean ± SEM for 5-6 rats. Data for the total number of feces and total number of diarrheal feces by H. odorata extracts, positive antidiarrheal control (Loperamide, 5 mg/kg p.o.) and normal control (saline) were processed by paired *t*-test analysis using Statistical package for social Science (SPSS for windows, version 21.0, IBM Corporation, New York, USA) followed by Dunnet test. ^a, b, c^ & ^d^ superscript letters in the table are statistically significant to each other. Values with *P* < 0.05 are considered as statistically significant

### Castor oil-induced enteropooling

As demonstrated in Table 3, all test doses of the extract significantly reduced the intestinal weight and volume in dose dependent manner. Castor oil caused accumulation of water and electrolytes in intestinal loop. Treatment with the *H. odorata* leave extracts (200 and 400 mg/kg) produced a significant (*P* < 0.05) inhibition of 45.57 ± 1.75%, 46.23 ± 0.96% and 38.03 ± 2.81% of intestinal content respectively in MEHO, EEHO and AEHO at 400 mg/kg body weight comparable to that of the standard drug loperamide which showed 59.70 ± 2.99% inhibition of intestinal content (Table 4).

**Table 3:** The effect of *H. odorata* extracts on castor oil induced enteropooling in mice

Treatment/Dose	Volume of intestinal content (ml)	Weight of intestinal content (g)	% Inhibition of intestinal content
Saline	0.24 ± 0.013	0.628 ± 0.033	
Loperamide	0.13 ± 0.001	0.316 ± 0.002^a^	48.2 ± 0.40
MEHO200	0.17 ± 0.007	0.396 ± 0.016^b^	35.08 ± 2.67
MEHO400	0.14 ± 0.005	0.332 ± 0.010^a^	45.57 ± 1.75
EEHO200	0.17 ± 0.004	0.388 ± 0.011^b^	36.39 ± 1.83
EEHO400	0.14 ± 0.003	0.328 ± 0.006^a^	46.23 ± 0.96
AEHO200	0.19 ± 0.004	0.428 ± 0.009^b^	29.84 ± 1.41
AEHO400	0.16 ± 0.007	0.378 ± 0.017^a^	38.03 ± 2.81

Values are presented as mean ± SEM for 5-6 rats. Data for the weight of intestinal content (g) by H. odorata extracts, positive antidiarrheal control (Loperamide, 5 mg/kg p.o.) and normal control (saline) were processed by paired *t*-test analysis using Statistical package for social Science (SPSS for windows, version 21.0, IBM Corporation, New York, USA) followed by Dunnet test. ^a^ & ^b^ superscript letters are statistically significant to each other. Values with *P* < 0.05 are considered as statistically significant

**Table 4:** The effect of *H. odorata* extracts on intestinal transit in mice using charcoal meal as a marker

Treatment/Dose	% of intestine cross by marker	% of inhibition
Saline	84.85 ± 2.88	
Loperamide	45.35 ± 1.72^a^	43.6 ± 2.14
MEHO200	59.89 ± 0.51^b^	25.63 ± 0.63
MEHO400	52.72 ± 1.11^b^	34.43 ± 1.38
EEHO200	57.15 ± 0.81^b^	28.1 ± 1.01
EEHO400	51.51 ± 0.97^b^	35.93 ± 1.21
AEHO200	62.05 ± 1.41^b^	22.82 ± 1.76
AEHO400	55.04 ± 1.54^b^	31.54 ± 1.91

Values are presented as mean ± SEM for 5-6 rats. Data for % of intestine cross by H. odorata extracts, positive antidiarrheal control (Loperamide, 5 mg/kg p.o.) and normal control (saline) were processed by paired *t*-test analysis using Statistical package for social Science (SPSS for windows, version 21.0, IBM Corporation, New York, USA) followed by Dunnet test. ^a ^&^ b^ superscript letters are statistically significant to each other. Values with *P* < 0.05 are considered as statistically significant

### Gastrointestinal motility

The effect of *H. odorata* extract on the intestinal transit is depicted in Table 4. All doses of the extracts were successful to produce significant alteration in the % intestinal motility compared to the normal control. The normal control (saline) resulted in 84.85 ± 2.88% intestinal motility by the marker-charcoal meal. The 200 and 400 mg/kg oral dose of the extracts exhibited 51.51 ± 0.97% to 62.05 ± 1.41% intestinal motility (Table 5). And the extracts significantly inhibited 22.82 ± 1.76% to 35.93 ± 1.21% of intestinal motility at all the doses. However, the standard drug, Loperamide (5 mg/kg) demonstrated a significant inhibition (43.6 ± 2.14%) in intestinal motility.

**Table 5:** Effect of *H. odorata* extracts on fasting blood glucose level (mmol/L) in normal mice

Group	Dose (oral)	Before administration (mmol/L)	After administration (mmol/L)
Control (water)	10 ml/kg	3.48 ± 0.11	4.29 ± 0.26
Glibenclamide	5 mg/kg	4.04 ± 0.09^a^	2.70 ± 0.18^a^
MEHO	800 mg/kg	4.48 ± 0.36	6.26 ± 0.31^a^
EEHO	800 mg/kg	4.54 ± 0.22^a^	5.98 ± 0.12^a^
AEHO	800 mg/kg	4.52 ± 0.11^b^	5.30 ± 0.09

Values are presented as mean ± SEM for 5-6 rats. Data for blood glucose concentrations were processed by paired *t*-test analysis by using Statistical Package for Social Science (SPSS for windows, version 21.0, IBM Corporation, New York, USA) followed by Dunnet test. ^a ^&^ b^ superscript letters are statistically significant to each other. Values with *P* < 0.05 are considered as statistically significant

### Hypoglycemic effect

Data for hypoglycemic effects of *H. odorata* are presented in Table 5. At 800 mg/kg dose of *H. odorata* extracts leave showed no hypoglycemic activity, i.e. they didn’t reduce fasting blood glucose level rather fasting blood glucose level was somewhat increased with the administration of *H. odorata* extracts. However, glibenclamide significantly (*P* < 0.01) reduced fasting blood glucose level.

### In vitro thrombolysis

**Fig. 3:**
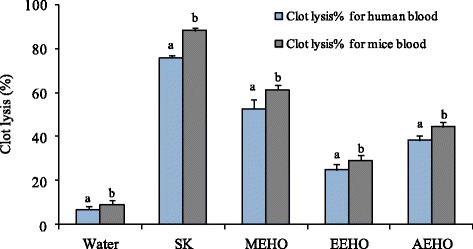
Comparative clot lysis by the controls and *H. odorata* extracts on both human and mice blood. Values are expressed as mean ± SEM. Data for clot lysis percentage were processed by paired *t*- test using Statistical package for social Science (SPSS for windows, version 21.0, IBM Corporation, New York, USA) followed by Dunnett test. ^a^ &^ b^ superscript letters on the graph bars are significantly different from each other. Values with *P* < 0.05 were considered as significant

Table 6 summarizes the thrombolytic effects of *H. odorata* extracts both on human and animal blood. In thrombolytic approach with human blood sample, addition of 100 *μ*L streptokinase showed 76 ± 1.13% clot lysis. On the other hand, distilled water was treated as negative control which showed only 6.8 ± 1.38%, a negligible clot lysis. MEHO showed the highest significant (52.88 ± 4.1%) clot lysis activity among all the extracts (*P* < 0.001). EEHO and AEHO respectively showed 24.92 ± 2.44% and 38.25 ± 2.25% of clot lysis. Percentages of clot lysis obtained after treating the clots with different extracts and appropriate controls are shown in Table 7 and their comparison was represented in Fig. 3.

**Table 6:** In vitro clot lysis activity of *H. odorata* extracts and Streptokinase on human and mice blood

Drug/Extracts	% of clot lysis for human blood	*P* value (two-tailed) when compared to negative control	% of clot lysis for mice blood	*P* value (two-tailed) when compared to negative control
H_2_O	06.80 ± 1.38	-	9.06 ± 1.76	
SK	76.00 ± 1.13^c^	*P* < 0.001	88.25 ± 1.42^c^	*P* < 0.001
MEHO	52.88 ± 4.1^c^	*P* < 0.001	61.34 ± 2.23^c^	*P* < 0.001
EEHO	24.92 ± 2.44^a^	*P* < 0.05	29.16 ± 2.58^c^	*P* < 0.001
AEHO	38.25 ± 2.25^c^	*P* < 0.001	44.48 ± 2.04^b^	*P* < 0.01

Values are mean ± SEM for 20 volunteers. Data for the effective clot lysis percentage by extracts, positive control (streptokinase) and normal control (distilled water) were processed by paired t-test analysis using Statistical package for social Science (SPSS for windows, version 21.0, IBM Corporation, New York, USA) followed by Dunnet test. ^a, b^ & ^c^ superscript letters are statistically significant to each other. Values with *P* < 0.05 are considered as statistically significant

**Table 7:** PASS prediction data biological activities of Balanocarpol, Hopeaphenol and Ampelopsin H

Biological activity			PASS predictions			
	Balanocarpol		Hopeaphenol		Ampelopsin H	
	Pa	Pi	Pa	Pi	Pa	Pi
Antioxidant	**0.597**	0.005	0.515	0.006	0.497	0.007
Free radical scavenger	0.616	0.005	0.549	0.008	0.504	0.01
Nitric oxide scavenger	**0.227**	0.031	0.221	0.035	0.221	0.035
Cardioprotectant	0.289	0.092	**0.294**	0.088	**0.294**	0.088
Hepatoprotectant	**0.379**	0.036	0.369	0.038	0.369	0.038
Thrombolytic	0.692	0.042	**0.730**	0.038	0.708	0.051
Fibrinolytic	0.675	0.033	**0.690**	0.026	**0.690**	0.026
Antibacterial	**0.578**	0.006	0.313	0.097	0.446	0.066
Antifungal	**0.466**	0.004	0.250	0.106	0.234	0.116
Anticarcinogenic	**0.627**	0.011	0.470	0.022	0.431	0.026
Antihelmintic	0.185	0.107	**0.331**	0.081	**0.331**	0.081
Antiinflammatory	0.239	0.226	0.274	0.188	**0.286**	0.175

Bold text indicates the highest probability of activity (Pa) for each biological activity

Methanol extract of *H. odorata* (MEHO) showed the highest significant (61.34 ± 2.23%) clot lysis activity among all the extracts (*P* < 0.001) and the value was statistically significant compared to that of Streptokinase which showed 88.25 ± 1.42% clot lysis. EEHO showed 29.16 ± 2.58% of clot lysis and its *P* value was 0.001 and AEHO showed 44.48 ± 2.04% of clot lysis and its *P* value was 0.01. Percentages of clot lysis obtained after treating the clots with different extracts and appropriate controls are shown in Table 7 and their comparison was represented in Fig. 3.

### In silico PASS prediction

Three phytoconstituents namely Balanocarpol, Hopeaphenol and Ampelopsin H were analyzed by the PASS for their different types of biological activity and results were used in a flexible manner. All the compounds showed greater Pa than Pi (Table 7). Balanocarpol showed highest Pa for almost seven biological activities at range 0.227–0.627 and showed near highest Pa value for other five biological activities at range 0.239–0.692. But it exhibited low Pa value for anthelmintic activity. Hopeaphenol and Ampelopsin H exhibited same Pa and Pi value for some biological activity. Pa and Pi values for different activities of examined phytoconstituents are presented in Table 7.

## Discussion

Medicinal plants have recently drawn much attention of the scientists due to the strong healing effect with no or less adverse effects. The present investigation has explored the use of a medicinal plant (*H. odorata*) as antioxidant, antidiarrheal, hypoglycemic and thrombolytic properties. Antioxidants fight against free radicals playing a definite role in numerous pathological demonstrationsand protects us from different diseases. They exert their action either by scavenging the reactive oxygen species or protecting the antioxidant defense mechanisms [45]. The most natural antioxidants are multifunctional. Therefore, a reliable antioxidant evaluation protocol requires different antioxidant activity assessments to account various mechanisms of antioxidant action.

Polyphenols were found in all the extracts of *H. odorata* leaves. These polyphenolics are important dietary antioxidants because they have ideal chemical structures for free radical scavenging activities, and have been shown to be more effective antioxidants in vitro than vitamins E and C on a molar basis [46]. Our findings suggested that leaves of *H. odorata* rich in phenolic and flavonol contents which are the major contributor to scavenge the free radicals in oxidation pathways.

The reducing power of all extracts was determined by direct electron donation in the reduction of ferri cyanide [Fe(CN)_6_]^3-^ to ferro cyanide [Fe(CN)_6_]^4-^. The presence of reductant (i.e. antioxidants) in *H. odorata* leaves cause the reduction of the Fe^3+^ /ferricyanide complex to the ferrous form which was monitored by measuring the formation of Perl’s Prussian blue at 700 nm. The highest reducing power of MEHO at all the tested concentrations might be due to the phenolic compounds, especially Balanocarpol, Hopeaphenol and Ampelopsin H the major compounds of *H. odorata*, playing pivotal role in reducing power of the extracts. [47].

Hydrogen peroxide scavenging assay is one of the promising biologically important antioxidative assays. H_2_O_2_ is rapidly decomposed into oxygen and water producing hydroxyl radicals (^**•**^OH) that can initiate lipid peroxidation and cause DNA damage [48]. Methanol extract of *H. odorata* efficiently scavenged hydrogen peroxide which may be attributed to the presence of phenolic groups that could donate electrons to hydrogen peroxide, thereby neutralizing it into water.

In antidiarrheal screening, castor oil is known to cause water and electrolyte permeability changes in the intestinal mucosal membranes, resulting in fluid and watery luminal content that flow rapidly through the small and large intestines [49]. The induction of diarrhea by castor oil is attributed to its active ingredient ricinoleic acid which stimulates the production of several inflammatory mediator substances that include prostaglandins, nitric oxide, and platelet activating factor, cAMP, histamine and tachykinins [50]. The mediators thus released promote vasodilatation, smooth muscle contraction, and mucus secretion in the small intestines. The inhibition of castor-oil induced diarrhea can therefore be linked with the inhibition of the synthesis of all or either of those mediators by the experimental extract. In the enteropooling study, the plant extract also dose-dependently and significantly inhibited all the diarrheal parameters (onset, frequency and severity of diarrhea, total number of stools, number of wet stools, weight of wet stools, etc.). This significant inhibition of enteropooling, therefore, suggests that the extract probably produces relief in diarrhea through its spasmolytic and anti-enteropooling effects. The intraluminal fluid accumulation induced by castor oil was also blocked by the extract in a dose-related manner. Although the exact mechanism of the antidiarrheal action of *H. odorata* could not be established in this study, a number of investigators have shown the flavonoids, triterpenoids, saponins, especially flavonoid possesses ability to inhibit intestinal motility and hydroelectrolytic secretions which are known to be altered in diarrheic conditions [51]. Since *H. odorata* is known to contain high content of resveratrol derivatives those are stilbenoid and obtained from same substrate of flavonoid giving very strong antioxidative character, it is not unreasonable to speculate that they could have contributed to the observed antidiarrheal effect of the plant.

In the hypoglycemic activity test extracts of *H. odorata*exerted no hypoglycemic effect after 2 h of administration implying the absence of the extract metabolites could function to lower the blood glucose level at least in this experiment. Glibenclamide, stimulator of insulin secretion from β-cells islets of Langerhans, significantly reduced the blood glucose level but no significant glucose lowering effect was observed for the fractions of *H. odorata* demonstrating that the beta cells were not sensitized enough to secret insulin from β-cells by the extract.

Thrombolytic drugs block the pathway of thrombus formation with the help of plasmin that lyses clot by breaking down the fibrinogen and fibrin contained in a clot. Commercial thrombolytic drugs especially streptokinase converts plasminogen to plasmin through the and thereby increases clot lysis. Scientists reported that flavonoids, among the plant metabolites, affect thrombosis and cardiovascular disease by interfering with platelet activation which a potential risk factor for cardiovascular disease [52]. High content of *H. odorata* is very much consistent with the previous report implying the role of *H. odorata* especially MEHO in clot lysis. Additionally the comparison of positive control with negative control clearly demonstrated that clot lysis does not occur with the addition of water in our experiment.

The PASS prediction test for Balanocarpol, Hopeaphenol and Ampelopsin H isolated from *H. odorata* showed very good prospects and possibilities for thrombolytic activity with wide range of Pa value as 0.662–0.730. It is reported that there are bacterial contaminants of plants which have plasminogen receptors that bind plasminogen. Cell surface bound plasminogen is easily activated to plasmin, which could lead to fibrinolysis [53]. *H. odorata* has moderate antibacterial activity [54]. Bacterial plasminogen activator: staphylokinase, streptokinase, act as cofactor molecules that contribute to exosite formation and enhance the substrate presentation to the enzyme. Staphylokinase activates plasminogen to dissolve clots, also destroys the extracellular matrix and fibrin fibers that hold cells together [55]. However the thrombolysis on human and mice blood differs, which is expected, due to the significant differences between mice and humans in immune system development, activation, and response to challenge, in both the innate and adaptive arms. The differences are quite helpful to validate the experimental procedure, technical drawbacks and efficacy of the therapeutics.

In order to accelerate the research for potent natural products, computer-aided drug discovery program PASS was used to predict the biological activity. PASS prediction tools were constructed using 20,000 principal compounds [42]. The result of prediction is presented as the list of activities with appropriate Pa and Pi ratio. In the prediction of biological activity of the examined phytoconstituents, Balanocarpol showed maximum Pa value 0.227–0.627 for antioxidant, free radical scavenger, nitric oxide scavenger, hepatoprotectant, antibacterial, antifungal and anticarcinogenic. Hopeaphenol and Ampelopsin H both exhibited maximum probability for cardioprotectant (Pa = 0.294), fibrinolytic (Pa = 0.690) and antihelmintic (Pa = 0.331). Alone Hopeaphenol exhibited maximum probability for thrombolytic (Pa = 0.730) and ampelopsin H for anti-inflammatory (Pa = 0.286) action.

## Conclusions

These findings suggest that the plant may be a potential source for the development of new antidiarrheal drug. Also data from present results revealed that *H. odorata* act as an antioxidant agent due to its H_2_O_2_ scavenging and thrombolytic activity. Though this plant has not been found to be hypoglycemic in normoglycemic model, it may be further studied in diabetic model to confirm. Further research may result *H. odorata* as good source of natural antioxidant, antidiarrheal and thrombolytic drug. PASS prediction matched with present study for the extracts. So further study need to identify the activity as PASS predicted for the phytoconstituents.

## Additional file

Additional file 1: Cover letter and author's form clarifying the revision of the manuscript and inclusion of an author. (DOC 118 kb)

## Abbreviations

AA: Ascorbic acids
AEHO: Water extract
EEHO: Ethanol extract
GAE: Gallic acid equivalent
MEHO: Methanol extract of *H. odorata*
QE: Quercetin equivalent
SEM: Standard error for mean
