# Supplementary material for: Antioxidant, antidiarrheal, hypoglycemic and thrombolytic activities of organic and aqueous extracts of Hopea odorata leaves and in silico PASS prediction of its isolated compounds
Source: BMC Complement Altern Med. 2016 Nov 21;16:474. doi: 10.1186/s12906-016-1461-x (PMC5117591; doi:10.1186/s12906-016-1461-x)
Supplement: Additional file 1: — Cover letter and author's form clarifying the revision of the manuscript and inclusion of an author. (DOC 118 kb) [file 12906_2016_1461_MOESM1_ESM.doc]

**Please read the important information on page 4 before you begin**

This form should be used by authors to request any change in authorship. Please fully complete all sections. Use black ink and block capitals and provide each author’s full name with the given name first followed by the family name.

**Section 1 Please provide the current title of manuscript**

**Manuscript ID no.** BCAM-D-16-00831

**Antioxidant, antidiarrheal, hypoglycemic and thrombolytic activities of organic and aqueous extracts of *Hopea odorata* leaves and in silico PASS prediction of its isolated compounds**

**Section 2 Please provide the current authorship, in the order shown on your manuscript.**

Please use an additional sheet if there are more than 7 authors.

|  | First name(s) | Family name |
| --- | --- | --- |
| 1^st^ author | Mohammad Shah Hafez | Kabir |
| 2^nd^ author | Mohammed Munawar | Hossain |
| 3^rd^ author | Md. Imtiazul | Kabir |
| 4^th^ author | Shabbir | Ahmad |
| 5^th^ author | Nishan | Chakrabarty |
| 6^th^ author | Md. Atiar | Rahman* |
| 7^th^ author |  |  |

**Section 3: Please provide a justification for change. Please use this section to explain your reasons for changing the authorship of your manuscript. Please refer to the journal policy pages for more information about authorship. Please explain why omitted authors were not originally included on the submitted manuscript.**

**The proposed author** made substantial contributions to design the work and interpretation of data. He is also agreed to be accountable for all aspects of the work in ensuring that questions related to the accuracy or integrity of any part of the work are appropriately investigated and resolved. We have included the author in our first submission but during the revision procedures we asked one of our coauthors to revise the manuscript and he mistakenly did it. But when the author noticed that he is not receiving message from BMC CAM, I came to know and addressed accordingly.

**Section 4** Proposed new authorship. Please provide your new authorship list in the order you would like it to appear on the manuscript.

|  | First name(s) | Family name (this name will appear in full on the final publication and will be searchable on PubMed and similar databases) |
| --- | --- | --- |
| 1^st^ author | Mohammad Shah Hafez | Kabir |
| 2^nd^ author | Mohammed Munawar | Hossain |
| 3^rd^ author | Md. Imtiazul | Kabir |
| 4^th^ author | Shabbir | Ahmad |
| 5^th^ author | Nishan | Chakrabarty |
| 6^th^ author | Md. Mominur | Rahman |
| 7^th^ author | Md. Atiar | Rahman* |
|  |  |  |

Please use an additional sheet if there are more than 7 authors.

**Section 5 Author contribution, Acknowledgement and competing interests section. Please use this section to provide revised Author Contribution, Acknowledgement and/or Competing Interests sections of your manuscript, ensuring you state what contribution any new authors made and, if appropriate acknowledge any contributors who have been removed as authors. Please ensure these are updated in your manuscript.**

**New Competing Interests statement:**

The authors declare that they have no competing interests

**New Author Contributions statement:**

This work was carried out in collaboration between all authors. Authors MSHK, MMH and AH collected the plant leaves. MSHK carried out the study design, data collection, data interpretation, manuscript preparation, statistical analysis and performed PASS prediction. MSHK, MMH, SA and NC participated in experimental works, data collection and literature search. MMR supervised the study design and data interpretation. Author MAR contributed in research design, data interpretation, manuscript preparation and statistical analysis. MAR also acted for all correspondences. All authors read and approved the final version of the manuscript.

**New Acknowledgement Section:**

The authors are grateful to the authority of International Islamic University Chittagong, Bangladesh, for providing the facilities to conduct this research work. The authors are also thankful to Dr. Sheikh Bokhtear Uddin, Taxonomist and Professor, Department of Botany, University of Chittagong, for identifying the plant. Authors also wish to thank Tanvir Ahmad Chowdhury, Abul Hasanat, Md. Akramul Hoque of International Islamic University to help collect plant samples.

**State ‘Not applicable’ if there are no new authors.**

**Section 6 Declaration of agreement. *All* authors, unchanged, new and removed *must* sign this declaration.**

*** please delete as appropriate. Delete all of the bold if you were on the original authorship list and are remaining as an author**

|  | First name | Family name |  | Signature | Affiliated institute | Date |
| --- | --- | --- | --- | --- | --- | --- |
| 1^st^ author | Mohammad Shah Hafez | Kabir | I agree to the proposed new authorship shown in section 4 /**and the addition/removal*****of my name to the authorship list.** |  | Department of Pharmacy, International Islamic University Chittagong, Bangladesh | 11 Nov 2106 |
| 2^nd^ author | Mohammed Munawar | Hossain | I agree to the proposed new authorship shown in section 4 /**and the addition/removal*****of my name to the authorship list.** |  | Department of Pharmacy, International Islamic University Chittagong, Bangladesh | 11 Nov 2106 |
| 3^rd^ author | Md. Imtiazul | Kabir | I agree to the proposed new authorship shown in section 4 /**and the addition/removal*****of my name to the authorship list.** |  | Department of Pharmacy, International Islamic University Chittagong, Bangladesh | 11 Nov 2106 |
| 4^th^ authors | Shabbir | Ahmad | I agree to the proposed new authorship shown in section 4 /**and the addition/removal*****of my name to the authorship list.** |  | Department of Pharmacy, International Islamic University Chittagong, Bangladesh | 11 Nov 2106 |
| 5^th^ author | Nishan | Chakrabarty | I agree to the proposed new authorship shown in section 4 /**and the addition/removal*****of my name to the authorship list.** |  | Department of Pharmacy, International Islamic University Chittagong, Bangladesh | 11 Nov 2106 |
| 6^th^ author | Md. Mominur | Rahman | I agree to the proposed new authorship shown in section 4 /**and the addition/removal*****of my name to the authorship list.** |  | Department of Pharmacy, International Islamic University Chittagong, Bangladesh | 11 Nov 2106 |
| 7^th^ author | Md. Atiar | Rahman* | I agree to the proposed new authorship shown in section 4 /**and the addition/removal*****of my name to the authorship list.** |  | Department of Biochemistry and Molecular Biology, University of Chittagong, Chittagong-4331, Bangladesh | 11 Nov 2106 |

Please use an additional sheet if there are more than 7 authors. * please delete as appropriate. Delete all of the bold if you were on the original authorship list and are remaining.

**Important information. Please read.**

- Please return this form, fully completed, to the editorial office. We will consider the information you have provided to decide whether to approve the proposed change in authorship. We may choose to contact your institution for more information or undertake a further investigation, if appropriate, before making a final decision.
- Please note, we cannot investigate or mediate any authorship disputes. If you are unable to obtained agreement from all authors (included those who you wish to be removed) you must refer the matter to your institution(s) for investigation. Please inform us if you need to do this.
- If you are not able to return a fully completed form within **14 days** of the date that it was sent to the author requesting the change, we may have to reject your manuscript. We cannot publish manuscripts where authorship has not been agreed by all authors (including those who have been removed).
- Incomplete forms will be rejected.
